# Indoline-6-Sulfonamide Inhibitors of the Bacterial Enzyme DapE

**DOI:** 10.3390/antibiotics9090595

**Published:** 2020-09-11

**Authors:** Cory T. Reidl, Tahirah K. Heath, Iman Darwish, Rachel M. Torrez, Maxwell Moore, Elliot Gild, Boguslaw P. Nocek, Anna Starus, Richard C. Holz, Daniel P. Becker

**Affiliations:** 1 Department of Chemistry and Biochemistry, Loyola University Chicago, 1032 West Sheridan Road, Chicago, IL 60660, USA; cory.reidl@northwestern.edu (C.T.R.); tkheath10@gmail.com (T.K.H.); idarwish@luc.edu (I.D.); torrezrm@med.umich.edu (R.M.T.); maxmoore@scripps.edu (M.M.); egild@luc.edu (E.G.); astarus@luc.edu (A.S.); 2 Department of Chemistry, Department of Molecular Biosciences, Chemistry of Life Processes Institute, Center for Molecular Innovation and Drug Discovery, Northwestern University, Evanston, IL 60208-3113, USA; 3 Midwest Center for Structural Genomics and Structural Biology Center, Biosciences Division, Argonne National Laboratory, Argonne, IL 60439, USA; boguslaw.nocek@abbvie.com; 4 Department of Chemistry, Colorado School of Mines, 1500 Illinois St., Golden, CO 80401, USA

**Keywords:** diaminopimelate desuccinylase, DapE, ninhydrin enzyme assay, indoline, sulfonamide, enzyme inhibition, antibiotic

## Abstract

Inhibitors of the bacterial enzyme dapE-encoded *N*-succinyl-l,l-diaminopimelic acid desuccinylase (DapE; EC 3.5.1.18) hold promise as antibiotics with a new mechanism of action. Herein we describe the discovery of a new series of indoline sulfonamide DapE inhibitors from a high-throughput screen and the synthesis of a series of analogs. Inhibitory potency was measured by a ninhydrin-based DapE assay recently developed by our group. Molecular docking experiments suggest active site binding with the sulfonamide acting as a zinc-binding group (ZBG).

## 1. Introduction

**Figure 1:**
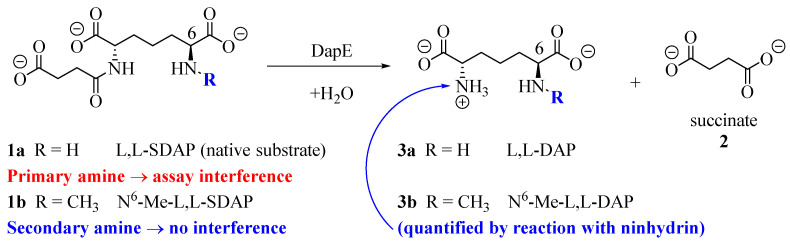
Hydrolysis of *N-*succinyl-l,l-diaminopimelic acid (l,l-SDAP) and *N*^6^-Me-SDAP by *Hi*DapE. L,L-SDAP (**1a**) and *N*^6^-methyl SDAP analog (**1b**) and with the formation of hydrolysis products succinate (**2**) and L,L-diaminopimelic acids (l,l-DAP) (**3a**,**b**).

Although momentarily eclipsed by the global COVID-19 pandemic, we continue to face a global healthcare crisis due to the increasing resistance of bacteria to all approved antibiotics. Invasive methicillin-resistant *Staphylococcus aureus* (MRSA) is a serious and growing health problem [1]. Newly discovered strains of MRSA show antibiotic resistance even to vancomycin, which has served as a drug of last resort for treating systemic infections [2]. Moreover, multi-drug resistant tuberculosis (TB), an infectious disease caused by the pathogen *Mycobacterium tuberculosis *(Mtb), continues to be a leading cause of death from an infectious agent worldwide. In 2018, the World Health Organization (WHO) estimated that 1.5 million of the approximately 10 million people who acquired a TB infection succumbed to this devastating chronic illness. [3,4] The steady increase in mortality and morbidity from bacterial infections by antibiotic-resistant bacteria [5] reveals the urgent need to discover antibiotics with new mechanisms of action. A very attractive and underexplored bacterial enzyme target is the *dapE*-encoded *N-*succinyl-l,l-diaminopimelic acid desuccinylase (DapE, E.C. 3.5.1.18) [6] that is present in all Gram-negative and most Gram-positive bacteria. DapE is one of the key enzymes in the lysine biosynthetic pathway in bacteria responsible for the biosynthesis of lysine and *meso*-diaminopimelate (*m*-DAP) [7], that are both essential for cell-wall synthesis. The enzyme DapE catalyzes the hydrolysis of *N*-succinyl-l,l-diaminopimelic acid (l,l-SDAP) to succinate and l,l-diaminopimelic acid (l,l-DAP) (Figure 1). The deletion of the DapE gene has been shown to be lethal to *Helicobacter pylori* and *Mycobacterium smegmatis*, demonstrating the indispensable role of this enzyme in bacterial survival and pathogenesis [8,9]. Furthermore, the lack of a similar enzymatic pathway in humans suggests that inhibition of DapE would provide selective toxicity to bacteria-free mechanism-based side effects in humans, making DapE a very promising target for antibiotics with a new mechanism of action [6].

To conveniently measure the inhibitory potency of test compounds against DapE, we recently developed a ninhydrin-based assay employing the unnatural but well-tolerated substrate *N*^6^-methyl-l,l-SDAP (**1b**) [10]. Enzymatic cleavage of **1b** affords primary amine product **3b** (Figure 1), which can be quantified spectrophotometrically following treatment with ninhydrin. Importantly, this assay avoids the significant background signal that would be produced by the reaction of the primary amine in native substrate **1a** with ninhydrin.

The first X-ray crystal structure of apo-DapE, lacking active-site zinc atoms, was solved in 2005 [11] and was followed by structures of mono- and di-Zn forms from *Haemophilus influenzae* [12] as well as mono- and di-Zn forms from *N. meningitidis* [13] In sharp distinction to the reported open-DapE structures, some of us recently reported a new X-ray crystal structure of DapE revealing the heretofore-unknown closed conformation of dimeric DapE containing the products of enzymatic cleavage, succinate, and diaminopimelic acid, bound in the active site [14]. This structure uncovered the role of His194B of the opposite peptide chain in the dimeric enzyme, which moves 10.1 Å to provide a key H-bond in the active site, activating the substrate for enzymatic cleavage [14]. This new insight into the enzymatic mechanism also explains the observed inactivity of monomeric DapE constructs [10]. This products-bound DapE structure has enabled further refinement of a mechanistic hypothesis for amide bond cleavage by DapE enzymes, supported by our products-bound transition state modeling (PBTSM) approach [14,15], which in turn will facilitate inhibitor identification. 

Earlier, we screened a small, focused library of compounds containing zinc-binding groups that led to the identification of the thiol-containing angiotensin-converting enzyme (ACE) inhibitor captopril as a low micromolar competitive inhibitor (IC_50_ = 3.3 μM) of DapE from *Haemophilus influenza* (*Hi*DapE) [16]. We then reported a high-resolution (1.8 Å) X-ray crystal structure of captopril bound to the active site of DapE from *Neisseria meningitidis* (*Nm*DapE), revealing the dinuclear Zn(II) metals bridged by the thiolate of captopril [13]. This structure has served as a model for in silico approaches to designing potential new inhibitors of DapE [17]. 

## 2. Results

**Figure 2:**
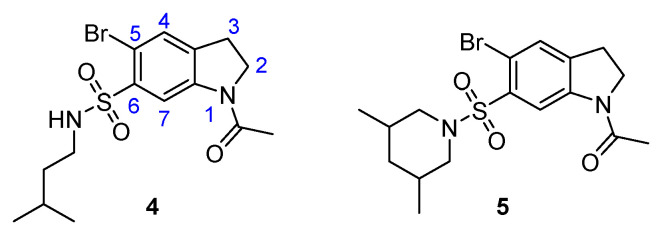
*N*-Acetyl-6-sulfonamide indoline screening hits from the high-throughput screen (HiTS).

We now report a high-throughput screen of ~33,000 compounds, which resulted in the discovery of two structurally similar indoline sulfonamide derivatives **4** and **5** (Figure 2) as inhibitors of DapE, showing >20% inhibition at 12 μM that was selected as an arbitrary cutoff. We were pleased that these two indoline hit structures provided mutual confirmation of one another. Furthermore, indolines are a privileged scaffold in medicinal chemistry, as there are several examples of indoline-containing drugs approved for a variety of treatments: advanced or transitional cell carcinoma of the urothelial tract (vinflunine) [18], glaucoma and severe anticholinergic toxicity (physostigmine) [19], and schizophrenia in adults (lumateperone) [20,21], or are under investigation, as for benign prostatic hyperplasia (BPH) [22]. Furthermore, sulfonamides are pervasive in agrochemicals and in pharmaceuticals across therapeutic areas [23,24,25,26,27], and primary sulfonamides have recently been leveraged as versatile intermediates [28].

### 2.1. Overview and Regiochemistry

Although the indoline sulfonamide hit structures were listed by the supplier as the corresponding indoline-7-sulfonamides, it was concluded that the compounds must be 6-sulfonamides (as illustrated in Figure 2), as there were no general methods in the literature to prepare the 7-sulfonamide hits. Specifically, electrophilic aromatic chlorosulfonylation of 5-bromo-*N*-acetyl-indoline affords the product of electrophilic aromatic substitution at the sterically preferred 6-position rather than the presumed 7-position, as clearly and definitively demonstrated by Borror [29]. Yet, widespread errors persist in the literature regarding the regiospecificity of sulfonylation of indolines. Herein, we report the synthesis and structure-activity relationships (SAR) of a series of 5-halo-6-indolinesulfonamides as new DapE inhibitors assessed using our new ninhydrin-based assay.

### 2.2. Molecular Docking Experiments

**Figure 3:**
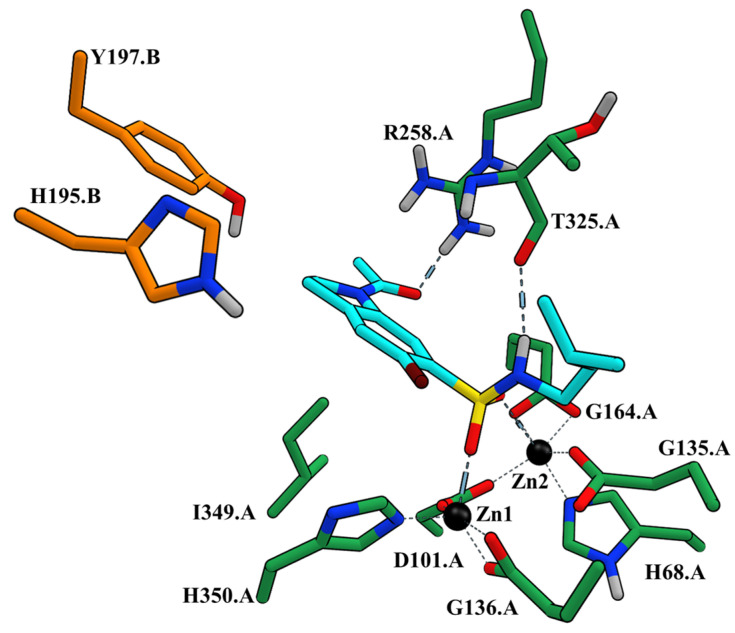
Docked N-isoamyl indoline sulfonamide hit compound **4** (cyan). The majority of the active site residues were contributed to by subunit I of the DapE dimer (green residues), but two residues came from subunit II of the dimer (orange).

Molecular docking employing the open crystal structure of DapE (PDB 5UEJ) was used to determine potential binding poses of inhibitors in the active site of the enzyme. Although the easily cloned and expressed *Hi*DapE enzyme was used in the assay, *Hi*DapE and *Nm*DapE share a very high sequence homology of 55% with no sequence gaps and bear the same active site architectures, including metal-binding residues and substrate-binding residues necessary for hydrolytic activity. We, therefore, decided to use the atomic-level resolution PDB 5UEJ structure of *Nm*DapE for docking. Docking of the indoline sulfonamide lead structures (**4** and **5**) suggests these compounds bind to the di-zinc active site indicative of competitive inhibition (Figure 3). Specifically, compound **4** docked to the active site of DapE, which suggests coordination of the two sulfonamide oxygen atoms with the two zinc atoms with simultaneous hydrogen bonding between the N-acetyl carbonyl and the guanidine moiety of Arg258A. Additionally, in this conformation, the proton on the nitrogen atom of the sulfonamide moiety is poised to form a strong hydrogen bond with the backbone carbonyl of Thr325A.

### 2.3. Chemistry

**Scheme 1:**
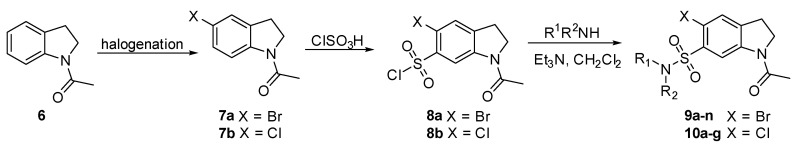
Synthesis of *N*-acetyl-5-bromo-6-sulfonamide indoline derivatives.

**Figure 4:**
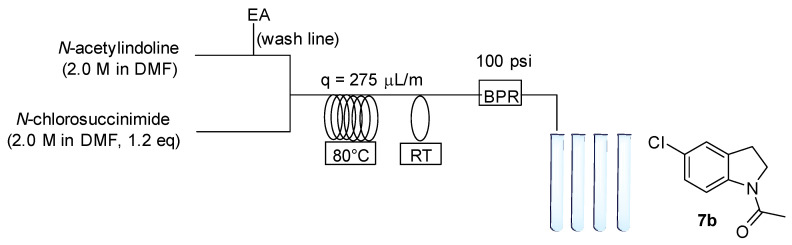
Flow chemistry apparatus for preparation of *N*-acetyl-5-chloroindoline **7b**.

Bromination of the *N*-acetyl indoline at the 5-position was performed by exposure of *N*-acetylindoline **6** to *N*-bromosuccinimide [29,30] in the presence of a catalytic amount of ammonium acetate to afford **7a** in an 83% yield (Scheme 1). Chlorination with *N*-chlorosuccinimide was more sluggish and provided the 5-chloroindoline derivative Figure S1 **7b** [31] in variable yield and required a tedious chromatographic purification. We, therefore, turned to a continuous flow synthesis condition for the synthesis of 5-chloroindoline derivative **7b** using conditions adapted from general flow methods [32], in particular, those developed by Pelleter [33]. Optimized continuous flow protocols afforded **7b** at a superior reaction rate and throughput, and provided excellent purity after direct crystallization of the product stream from water (Figure 4).

The 5-chloro and 5-bromo *N*-acetyl indoline analogs **7a** and **7b** were then subjected to chlorosulfonation at the 6-position by heating at 65–70 °C in neat chlorosulfonic acid. The reaction mixture was quenched over ice to afford the corresponding sulfonyl chlorides **8a** [29] and **8b**, respectively, in good purity based on nuclear magnetic resonance (NMR) spectroscopy, which were used directly in the next step.

A variety of primary and secondary alkyl amines were reacted with the 5-halo-6-sulfonyl chloride derivatives **8a** or **8b** in the presence of triethylamine base to afford a series of sulfonamide inhibitors **9a**–**n** and **10a**–**f**, respectively, according to general literature precedent [29,30]. In instances where the reacting amine was secondary, a catalytic amount of DMAP was added to the reaction solution. The sulfonamide products were isolated by extraction followed by passage through an activated carbon plug and recrystallization from chloroform–ethyl acetate to afford the final inhibitors in high purity. 

### 2.4. DapE Enzyme Inhibition and Structure–Activity Relationships

The compounds synthesized along with their DapE inhibitory potencies measured using the *N*-Me-SDAP substrate are summarized in Table 1. We kept the *N*-acetyl group as well as the 5-halogen moiety constant in this first series while varying the sulfonamide *N*-substituent. The 5-bromo-6-isoamylsulfonamide hit **4** was synthesized and tested first to confirm the activity of the original hit, which was found to exhibit a modest but measurable 42% inhibition at 200 µM. The corresponding isobutyl derivative **9a** was less potent, with a 20% inhibition at 200 µM. Among other secondary sulfonamides synthesized, *N*-cyclohexyl derivative **9b** was somewhat more potent with an IC_50_ of 162 µM, whereas *N*-benzyl and *N*-*t*-butyl derivatives **9c** and **9d** showed 56% and 39% inhibition at 200 µM and 100 µM, respectively. Several derivatives had poor solubility, limiting the ability to test inhibition at higher concentrations. The addition of more polar ester functionalities provided the nearly inactive glycine ester **9e**, while the β-alanine ester **9f** was found to be moderately potent with an IC_50_ of 118 µM. Valine ester **9g** was more potent, with an IC_50_ of 82 µM, whereas the phenylalanine ester **9h** was less potent, with 61% inhibition at 200 µM. 

Among secondary sulfonamides examined in this work, the simplified piperidine analog **9i** was synthesized to eliminate the stereochemical complexity of dimethyl piperidine hit compound **5**. Piperidine sulfonamide **9i** exhibited an IC_50_ of 133 µM, with the IC_50_ of the corresponding pyrrolidine sulfonamide **9j**, improving to 97 µM. The bulkier and more rigid indoline sulfonamide **9k** was synthesized to take advantage of hydrophobic interactions in the active site and resulted in an IC_50_ of 86 µM. Conversely, the more flexible acyclic secondary sulfonamides **9l** and **9m** were significantly less potent, whereas *N*,*N*-diethyl sulfonamide **9n** revealed an IC_50_ of 99 µM comparable to **9j**.

To decrease the molecular weight and logP, several 5-chloro derivatives were synthesized and evaluated for DapE inhibitory potency. Gratifyingly, the 5-chloro-isoamylsulfonamide **10a** with an IC_50_ of 54 µM was ~5× more potent than the corresponding 5-bromo derivative **4**. The 5-chloro-*N*-cyclohexyl derivative **10b** exhibited comparable potency to the corresponding 5-bromo derivative **9b**. Pyrrolidine 5-chloro derivative **10c** with an IC_50_ of 44 µM was ~3× more potent than the corresponding 5-bromo derivative **9i**, but the trend was not maintained for 5-chloro pyrrolidine derivative **10d** with an IC_50_ of 172 µM, relative to 5-bromo derivative **9j** with an IC_50_ of 97 µM. The di-*n*-propyl derivative **10e** with an IC_50_ of 88 µM was again more potent than the 5-bromo derivative **9l**.

Direct comparison of inhibitory data for 5-chloro and 5-bromo analogs shows that the inclusion of a 5-chloro substituent generally increased the inhibition of DapE relative to the 5-bromo series, with the 5-chloro piperidine **10a** and 5-chloro isopentyl **10c** showing the two most potent IC_50_ values in the series at 54 μM and 44 μM, respectively. Additionally, while some 5-bromo analogs showed solubilities at the higher concentrations, water solubilities increased on average for the chloro derivatives, making them more attractive as potential drug candidates.

## 3. Summary

In summary, a series of *N*-acetyl-5-halo-6-sulfonamide indolines was designed, synthesized, and assayed for inhibition against DapE. The original lead inhibitor isopentyl sulfonamide **4** and hit analog piperidine sulfonamide **9i** were found to be the modest inhibitors, exhibiting IC_50_ values of >200 μM and 130 μM, respectively. Significantly higher potencies were realized by varying the *N*-substitution of the sulfonamide moiety. Moreover, replacing the aryl bromide substituent with the corresponding chloride led to noticeably increased inhibition against DapE for nearly all analogs tested, as well as more favorable solubility properties. In silico studies were used to rationalize the improved potency of several key compounds. The 5-chloro analogs of the most promising 5-bromo ligands will be synthesized in future studies for continued SAR exploration, as will as *N*-acyl sulfonamides and sulfonylurea derivatives thereof. Inclusion of water-solubilizing groups, such as carboxylates, which also bind tightly to Zn(II) atoms, and tertiary amines, which will allow the production of ammonium salts, will be pursued in future efforts. This study represents the first reported inhibitory data and SAR analysis of DapE with the newly reported ninhydrin-based enzymatic assay and will be used to inform drug design strategies in the development of DapE inhibitors as potential antibiotics with a new mechanism of action. 

## 4. Materials and Methods

### 4.1. General Experimental Methods

All reagents were used as purchased without further purification unless otherwise noted. All solvents were distilled before use. All reactions were performed under an inert atmosphere of nitrogen. For chromatography, silica gel 60A, 40−75 μm (200 × 400 mesh) was utilized, and aluminum-backed silica gel 200 μm plates were used for thin-layer chromatography (TLC). ^1^H (proton) NMR spectra were obtained employing either a 400 MHz or a 500 MHz spectrometer with tetramethylsilane (TMS) as the internal standard. NMR spectra were processed using the Mnova NMR software program by Mestrelab Research. The purity of all assayed compounds was confirmed to be ≥95% unless otherwise noted, as determined by high-performance liquid chromatography (HPLC) utilizing a mobile phase A comprises 5% acetonitrile B in water and a mobile phase B = 0.1% trifluoroacetic acid (TFA) in acetonitrile, employing a gradient of 60% B increasing to 95% over 10 min, holding at 95% B for 5 min, and then returning to 60% B and finally holding for 5 min. High-resolution mass spectra (HRMS) spectra were measured on a Time-of-Flight (TOF) instrument utilizing the method of electrospray ionization (ESI). High-resolution mass spectral (HRMS) data were obtained at the Integrated Molecular Structure Education and Research Center (IMSERC, Northwestern University, Evanston, IL, USA) on an Agilent 6210A TOF mass spectrometer in the positive ion mode coupled to an Agilent 1200 series high-performance liquid chromatography (HPLC) system. Data were processed using MassHunter software version B.04.00.

### 4.2. Molecular Docking Protocol

Molecular models were developed using the Molecular Operating Environment (MOE) computational suite’s Builder utility, followed by minimization in the gas phase using the MMFF94X1 force field. The X-ray crystal structure of *Nm*DapE was then uploaded into the MOE and prepared for docking using MOE’s Structure Preparation utility. This high-resolution DapE structure from *Neisseria meningitidis* (PDB 5UEJ, 1.30 Å resolution) was used as a docking receptor due to the high quality of refinement at the dimerization loops, which play an important role in substrate binding. The hydrogen-bonding network of the docking receptor was further optimized at pH 7.4 by automatically sampling different tautomer/protomer states using Protonate3D, which calculates optimal protonation states, including titration, rotamer, and “flips” using a large-scale combinatorial search. The substrate-binding cleft of chain A, which includes the dinuclear Zn(II) metal center, was surveyed using MOE’s Site Finder utility and populated with inactivated dummy atoms that define the docking location. Following preparation of the *Nm*DapE docking receptor model, an induced-fit molecular docking using the previously generated ligand conformation database of 4 was carried out with solvent atoms inactivated at the docking site specified by the dummy atoms populating the substrate-binding cleft of chain A of the docking receptor. The proxy triangle method with London ΔG scoring generated 50 data points, which were further refined using the induced fit method with GBVI/WSA ΔG scoring to obtain the top 30 docking results. The Amber12:EHT3 force field was used to perform these calculations. The top ligand docking pose is shown in Figure 3.

### 4.3. DapE Enzyme Inhibition

DapE inhibition was measured according to the method previously described [10]. In brief, the enzymatic activity of DapE was measured in triplicate at 570 nm by the Ruhemann’s purple complex formed through the reaction of the exposed primary amine and ninhydrin. To 175 µL of 50 mM HEPES (4-(2-hydroxyethyl)-1-piperazineethanesulfonic acid) buffer at pH 7.5 with 5 µL of 1µM DapE stock solution at 30 °C, 20 µL of 10 mM monomethyl SDAP ((2S,6S)-2-(3-carboxypropanamido)-6-(methylamino)heptanedioic acid) TFA salt was added. The reaction was allowed to proceed for 10 min and quenched by heating at 100 °C for 1 min and subsequently cooling on ice for 1 min. To the cooled reaction, 2% ninhydrin reagent in 100% DMSO (final volume 300 µL) was added and subsequently heated to 80 °C for 15 min. This was quenched by placing in ice water for 2 min, and the absorbance of an 80 µL aliquot was read at 570 nm via a microplate reader. These reactions were set as 100% standard enzyme activity of DapE.

### 4.4. Protein Expression and Purification

DapE enzymes were cloned, expressed, and purified according to the standard protocol as described previously for *Hi*DapE [12] and the DapE from *Neisseria meningitidis* (*Nm*DapE) [13]. The cell pellet was thawed, and the cells were disrupted by sonication. The resulting cell debris was pelleted by centrifugation at 15,000 rpm for 40 min. at 4 °C and the supernatant was loaded onto a column packed with HisTrap HP resin from GE Healthcare and washed with twenty-bed volumes of lysis buffer. The His6-tagged *Hi*DapE enzyme was then eluted with an elution buffer comprising 500 mM NaCl, 5% glycerol, 50 mM HEPES, pH 8.0, 250 mM imidazole, and 10 mM 2-mercaptoethanol. The His6-tag was then removed with His6-tagged tobacco etch virus (TEV) protease for 16 h at 4 °C in 50 mM HEPES, pH 8.0. The cleaved DapE protein was then concentrated with a Centricon (30,000-MW cutoff; Amicon) to a volume of 3 mL and purified on a HiLoad 16/600 Superdex 200 Prep Grade column (GE Healthcare, Marlborough, MA, USA). The resulting solution was mixed with 5 mL of His-Trap HP resin packed on a column to remove the remaining cleaved His6-tag, uncut protein, and the His6-tagged TEV protease, while the eluent containing *Hi*DapE was collected and washed with crystallization buffer (150 mM NaCl, 20 mM HEPES pH 8.0, and 1 mM TCEP) and concentrated to a concentration of ~20 mg/mL. 

### 4.5. Synthetic Organic Chemistry

#### 4.5.1. 1-Acetyl-5-Chloroindoline (**7b**)

According to a general procedure, [31] *N*-chlorosuccinimide (NCS, 1.74 g, 13.0 mmol) was added to a solution of *N*-acetylindoline 6 (2.00 g, 12.4 mmol) and NH_4_OAc (96.1 mg, 1.24 mmol) in acetonitrile (65.1 mL), slowly and stirred at room temperature under air and monitored by TLC. The completed reaction was partitioned between methylene chloride and washed with 10 mL water, 10 mL HCl (1M), 10 mL brine solution before drying over sodium sulfate and concentration afforded the 1-acetyl-5-chloroindoline 7b with spectral data matching the literature [34]. ^1^H NMR (500 MHz CDCl_3_): δ 8.13 (d, *J* = 8.5 Hz, 1H), 7.15 (d, *J* = 9.5 Hz, 1H), 7.14 (s, 1H), 4.07 (t, *J* = 8.0 Hz, 2H), 3.18 (t, *J* = 8.5 Hz, 2H), 2.22 (s, 3H). ^13^C NMR (125 MHz, CDCl_3_): δ 168.8, 141.8, 133.2, 128.6, 127.6, 124.7, 124.8, 117.9, 49.1, 28.0, 24.3.

#### 4.5.2. 1-Acetyl 5-Bromoindoline-6-Sulfonyl Chloride (**8a**)

Chlorosulfonic acid (18.7 mL) was added dropwise by an addition funnel to an oven-dried round-bottom flask containing *N*-acetyl-5-bromoindoline 7a (2.50 g, 10.4 mmol). The neat reaction was stirred at 60–70 °C for 3 h. The resulting black solution was cooled to room temperature and then quenched by pouring slowly over ice with stirring. An off-white precipitate was isolated via vacuum filtration, washed with deionized water, and dried in vacuo to provide **8a** (1.83 g, 52%) [35]. ^1^H NMR (500 MHz, Acetone-*d*_6_) δ 8.99 (s, 1H), 7.84 (t, *J* = 1.2 Hz, 1H), 4.34 (t, *J* = 8.7 Hz, 2H), 3.42 (t, *J* = 8.7 Hz, 2H), 2.24 (s, 3H). ^13^C NMR (75 MHz, DMSO-*d*_6_) δ 169.2, 146.0, 142.4, 135.1, 130.3, 117.1, 113.3, 49.2, 27.4, 284 24.7.

#### 4.5.3. 1-Acetyl-5-Chloroindoline-6-Sulfonyl Chloride (**8b**)

Chlorosulfonic acid (3.75 mL) was added to chloroindoline **7b** (500 mg, 2.56 mmol) dropwise via a pressure-equalizing funnel with stirring, and the solution was then heated to 60–70 °C for 3 h. The resulting black solution was cooled to room temperature and carefully quenched by pouring slowly over solid ice with stirring. The resulting light yellow precipitate was collected by vacuum filtration, washed with water (3 × 10 mL), and dried under vacuum to produce the desired sulfonyl chloride **8b** (368 mg, 49%). ^1^H NMR (500 MHz, DMSO-*d*_6_) δ 8.57 (s, 1H), 7.19 (s, 1H), 4.09 (t, *J* = 8.5 Hz, 2H), 3.11 (t, *J* = 8.5 Hz, 2H), 2.15 (s, 3H).

#### 4.5.4. General Procedure for the Synthesis of N-acetyl 5-Bromo-6-Sulfonamide Indolines (**4**, **9** and **10**)

To a stirred solution of sulfonyl chloride **8a** or **8b** (1 eq, 0.148 mmol) and triethylamine (1.25 eq, 26.0 μL, 0.185 mmol) in methylene chloride was added the requisite amine (1.25 eq, 0.185 mmol), and the reaction was stirred at room temperature until the reaction was deemed complete by TLC. The reaction was then diluted with methylene chloride, washed with water (1×), twice with 1 M HCl, and then once with brine. The solution was dried over Na_2_SO_4_, filtered, and concentrated under vacuum to provide the indoline sulfonamide products. Secondary amines and nitrogen heterocycles were reacted in the presence of 4-dimethylaminopyridine (DMAP) as a catalyst (10 mol%), and in those cases, the reactions proceeded more slowly than reactions employing primary amines. The synthesis of **4**, **9a**–**n**, and **10a**–**f** were accomplished using this general procedure.

#### 4.5.5. 1-Acetyl-5-Bromo-*N*-Isopentylindoline-6-Sulfonamide (**4**)

Light yellow solid (114 mg, 99%): mp 190–191 °C. ^1^H NMR (500 MHz, CDCl_3_) δ 8.88 (s, 1H), 7.48 (s, 1H), 5.04 (s, 1H), 4.13 (t, *J* = 8.6 Hz, 2H), 3.29–3.21 (m, 2H), 2.91 (d, *J* = 6.6 Hz, 2H), 2.23 (s, 3H), 1.61 (dt, *J* = 13.3, 6.8 Hz, 1H), 1.38 (q, *J* = 7.5 Hz, 2H), 0.84 (d, *J* = 6.6 Hz, 6H). ^13^C NMR (126 MHz, CDCl_3_) δ 168.9, 142.9, 137.9, 137.3, 130.7, 119.2, 112.9, 49.0, 41.7, 38.3, 27.6, 25.4, 24.1, 22.3. HRMS-ESI [M + H]^+^ calculated for C_15_H_22_BrN_2_O_3_S^+^, 388.04564; found, 388.04563.

#### 4.5.6. 1-Acetyl-5-Bromo-*N*-Isobutylindoline-6-Sulfonamide (**9a**)

Tan solid (93 mg, 84%): mp 215 °C (softens), 225.4–226.1 °C; ^1^H NMR (500 MHz, CDCl_3_) δ 8.86 (s, 1H), 7.47 (s, 1H), 5.13 (t, *J* = 6.4 Hz, 1H), 4.13 (t, *J* = 8.6 Hz, 2H), 3.25 (t, *J* = 8.6 Hz, 2H), 2.70 (t, *J* = 6.6 Hz, 2H), 2.23 (s, 3H), 1.74 (dq, *J* = 13.4, 6.7 Hz, 1H), 0.89 (d, *J* = 6.7 Hz, 6H). ^13^C NMR (126 MHz, CDCl_3_) δ 169.2, 143.1, 138.2, 137.7, 130.9, 119.2, 113.1, 51.0, 49.2, 46.1, 28.7, 27.8, 24.3, 20.2, 8.9. HRMS-ESI: [M + H]^+^ calculated for C_14_H_20_BrN_2_O_3_S^+^, 374.02993; found, 377.02998.

#### 4.5.7. 1-Acetyl-5-Bromo-*N*-Cyclohexylindoline-6-Sulfonamide (**9b**)

White crystalline solid (92.6 mg, 78%): mp 226–228 °C; ^1^H NMR (500 MHz, CDCl_3_) δ 8.85 (s, 1H), 7.40 (s, 1H), 4.95 (d, *J* = 7.6 Hz, 1H), 4.07 (t, *J* = 8.6 Hz, 2H), 3.18 (t, *J* = 8.6 Hz, 2H), 3.09 (dt, *J* = 9.4, 4.5 Hz, 1H), 2.17 (s, 3H), 1.70 (dd, *J* = 10.1, 4.7 Hz, 2H), 1.56 (dd, *J* = 11.2, 5.3 Hz, 2H), 1.46–1.39 (m, 2H), 1.15 (q, *J* = 9.0 Hz, 4H). ^13^C NMR (126 MHz, CDCl_3_) δ 168.9, 142.8, 139.6, 137.1, 130.7, 118.7, 113.1, 52.9, 49.0, 33.7, 29.7, 27.6, 25.2, 24.5, 24.1. HRMS-ESI: [M + Na]^+^ calculated for C_16_H_20_BrN_2_NaO_3_S^−^, 422.02809; found, 422.02998.

#### 4.5.8. 1-Acetyl-*N*-Benzyl-5-Bromoindoline-6-Sulfonamide (**9c**)

Yellow solid (184 mg, 81%): mp 205–208 °C. ^1^H NMR (500 MHz, CDCl_3_) δ 8.79 (s, 1H), 7.53 (s, 1H), 7.42–7.22 (m, 5H), 4.45 (s, 2H), 4.14 (t, *J* = 8.6 Hz, 2H), 3.26 (t, *J* = 8.6 Hz, 2H), 2.24 (s, 3H). 

#### 4.5.9. 1-Acetyl-5-Bromo-*N*-(Tert-Butyl)Indoline-6-Sulfonamide (**9d**)

Off-white solid (44.2 mg, 40%): mp 224.4 °C (softens), 235.7–236.1 °C; ^1^H NMR (500 MHz, CDCl_3_) δ 8.92 (s, 1H), 7.44 (s, 1H), 5.07 (s, 1H), 4.16–4.09 (m, 2H), 3.24 (t, *J* = 8.6 Hz, 2H), 2.23 (s, 3H), 1.23 (s, 9H). ^13^C NMR (126 MHz, CDCl_3_) δ 168.9, 142.9, 141.5, 137.0, 130.6, 118.3, 113.0, 54.9, 48.9, 30.2, 30.1, 27.5, 24.1. HRMS-ESI: (M + NH_4_) + calculated for C_14_H_20_BrN_2_O_3_S^+^, 392.0638; found, 392.0639. 

#### 4.5.10. 1-Acetyl-5-Bromoindolin-6-(Sulfonyl Glycine Methyl Ester) (**9e**)

Tan solid (105 mg, 93%): mp 179–182 °C; ^1^H NMR (500 MHz, CDCl_3_) δ 8.86 (s, 1H), 7.50 (s, 1H), 5.74 (t, *J* = 5.6 Hz, 1H), 4.13 (t, *J* = 8.6 Hz, 2H), 3.82 (d, *J* = 5.3 Hz, 2H), 3.68 (s, 3H), 3.25 (t, *J* = 8.7 Hz, 2H), 2.24 (s, 3H). ^13^C NMR (126 MHz, CDCl_3_) δ 169.2, 161.8, 143.0, 137.9, 137.7, 131.1, 119.1, 113.7, 49.2, 46.1, 44.6, 24.3, 8.9.

#### 4.5.11. Methyl 3-((1-Acetyl-5-Bromoindoline)-6-Sulfonamido)Propanoate (**9f**)

Off-white solid (117 mg, 97%): mp 190–193 °C; ^1^H NMR (500 MHz, CDCl_3_) δ 8.87 (s, 1H), 7.47 (s, 1H), 5.76 (d, *J* = 6.6 Hz, 1H), 4.11 (t, *J* = 8.6 Hz, 2H), 3.67 (s, 3H), 3.23 (t, *J* = 8.6 Hz, 2H), 3.17 (dd, *J* = 12.0, 6.5 Hz, 2H), 2.50 (t, *J* = 5.9 Hz, 2H), 2.21 (s, 3H). ^13^C NMR (126 MHz, CDCl_3_) δ 172.3, 168.9, 142.9, 138.1, 137.5, 130.8, 119.0, 113.1, 52.0, 49.0, 38.8, 33.9, 27.6, 24.1. HRMS-ESI: [M + Na]^+^ calculated for C_14_H_17_BrN_2_NaO_5_S^+^, 426.9934; found, 426.9937. 

#### 4.5.12. Methyl ((1-Acetyl-5-Bromoindolin-6-yl)Sulfonyl)Valinate (**9g**)

Tan solid (111 mg, 87%): mp 190.9–191.7 °C; ^1^H NMR (300 MHz, CDCl_3_) δ 8.80 (s, 1H), 7.46 (s, 1H), 5.70 (d, *J* = 9.4 Hz, 1H), 4.12 (t, *J* = 9.2 Hz, 2H), 3.83 (dd, *J* = 9.4, 5.2 Hz, 1H), 3.54 (s, 3H), 3.24 (dt, *J* = 11.7, 5.4 Hz, 2H), 2.23 (s, 3H), 2.17–1.99 (m, 1H), 0.92 (dd, *J* = 10.5, 6.7 Hz, 6H). ^13^C NMR (126 MHz, CDCl_3_) δ 171.3, 168.9, 142.7, 138.2, 137.4, 130.7, 118.6, 113.6, 61.5, 52.2, 48.9, 31.8, 27.6, 24.1, 18.8, 17.6. HRMS-ESI: [M + H]^+^ calculated for C_16_H_22_BrN_2_O_5_S^+^, 433.0427; found, 433.0426.

#### 4.5.13. Methyl ((1-Acetyl-5-Bromoindolin-6-yl)Sulfonyl)-L-Phenylalaninate (**9h**)

Tan solid (127 mg, 90%): mp 184.0–184.8 °C; ^1^H NMR (500 MHz, CDCl_3_) δ 8.82 (s, 1H), 7.41 (s, 1H), 7.29–7.16 (m, 3H), 7.14–7.08 (m, 2H), 5.68 (d, *J* = 8.0 Hz, 1H), 4.28 (dt, *J* = 8.2, 5.8 Hz, 1H), 4.11 (td, *J* = 8.7, 5.1 Hz, 2H), 3.56 (s, 3H), 3.26–3.18 (m, 2H), 3.09 (dd, *J* = 5.9, 3.9 Hz, 2H), 2.23 (s, 3H). ^13^C NMR (126 MHz, CDCl_3_) δ 170.8, 168.9, 142.7, 138.2, 137.3, 135.0, 130.8, 129.5, 128.6, 127.2, 118.6, 113.5, 60.4, 57.0, 52.4, 48.9, 39.5, 27.6, 24.1, 14.2. HRMS-ESI: [M + Na]^+^ calculated for C_20_H_21_BrN_2_NaO_5_S^+^, 503.0247; found, 503.0255.

#### 4.5.14. 1-Acetyl-5-Bromo-6-(Piperidin-1-Sulfonyl) Indoline (**9i**)

Off-white crystalline solid (114 mg, 70%): mp 212–214 °C; ^1^H NMR (500 MHz, CDCl_3_) δ 8.68 (s, 1H), 7.41 (s, 1H), 4.05 (t, *J* = 8.5 Hz, 2H), 3.25–3.20 (m, 4H), 3.17 (t, *J* = 8.5 Hz, 2H), 2.16 (s, 3H), 1.57 (t, *J* = 5.6 Hz, 4H), 1.48 (q, *J* = 5.6 Hz, 2H). ^13^C NMR (126 MHz, CDCl_3_) δ 169.0, 142.5, 137.7, 137.0, 131.4, 118.7, 113.8, 49.0, 46.8, 29.7, 27.5, 25.6, 24.1, 23.8. HRMS-ESI: [M + H]^+^ calculated for C_15_H_20_BrN_2_O_3_S^+^, 387.0373; found, 387.0373.

#### 4.5.15. 1-Acetyl-5-Bromo-6-(Pyrrolidin-1-Sulfonyl) Indoline (**9j**)

Light brown crystalline solid (98.8 mg, 90%): mp 238–240 °C; ^1^H NMR (500 MHz, CDCl_3_) δ 8.61 (s, 1H), 7.43 (s, 1H), 4.06 (t, *J* = 8.6 Hz, 2H), 3.43–3.36 (m, 4H), 3.17 (t, *J* = 8.6 Hz, 2H), 2.16 (s, 3H), 1.92 (s, 4H). ^13^C NMR (126 MHz, CDCl_3_) δ 169.0, 142.6, 138.2, 136.8, 131.5, 117.7, 113.8, 49.0, 48.2, 27.5, 25.8, 24.1. HRMS-ESI: [M + H]^+^ calculated for C_14_H_18_BrN_2_O_3_S^+^, 373.0216; found, 373.0216.

#### 4.5.16. 1-Acetyl-5-Bromo-6-(Indolin-1-Sulfonyl) Indoline (**9k**)

Dark brown solid (52 mg, 83.4% yield), mp 215–218°C. ^1^H NMR (500 MHz, CDCl_3_) δ 8.86 (s, 1H), 7.47 (s, 1H), 7.30 (d, *J* = 8.1 Hz, 1H), 7.16 (d, *J* = 7.4 Hz, 1H), 7.09 (t, *J* = 7.8 Hz, 1H), 6.93 (t, *J* = 7.5 Hz, 1H), 4.26 (t, *J* = 8.4 Hz, 2H), 4.10 (t, *J* = 8.6 Hz, 2H), 3.19 (dt, *J* = 17.3, 8.5 Hz, 4H), 2.21 (s, 3H). ^13^C NMR (126 MHz, DMSO-*d*_6_) δ 169.1, 138.5, 131.6, 127.6, 125.5, 123.4, 118.9, 113.8, 113.6, 50.9, 49.2, 29.9, 28.1, 27.9, 24.4. HRMS-ESI: [M + Na]^+^ calculated for C_18_H_17_BrN_2_NaO_3_S^+^, 443.0035; found, 443.0037.

#### 4.5.17. 1-Acetyl-5-Bromo-*N*,*N*-Dipropylindoline-6-Sulfonamide (**9l**)

Light brown solid (104 mg, 85%): mp 118–120 °C; ^1^H NMR (500 MHz, CDCl_3_) δ 8.71 (s, 1H), 7.49 (s, 1H), 4.13 (t, *J* = 8.6 Hz, 2H), 3.33–3.27 (m, 4H), 3.24 (t, *J* = 8.6 Hz, 2H), 2.24 (s, 3H), 1.62 (q, *J* = 7.6 Hz, 4H), 0.86 (t, *J* = 7.4 Hz, 6H). 

#### 4.5.18. 1-Acetyl-5-Bromo-*N*,*N*-bis(2-Methoxyethyl)Indoline-6-Sulfonamide (**9m**)

Tan solid (125 mg, 97%): mp 110–111 °C; ^1^H NMR (500 MHz, CDCl_3_) δ 8.78 (s, 1H), 7.49 (s, 1H), 4.23 (t, *J* = 8.5 Hz, 2H), 3.40 (t, *J* = 6.0 Hz, 4H), 3.53 (t, *J* = 5.5 Hz, 4H), 3.30 (s, 6H), 3.24 (t, *J* = 9.0 Hz, 2H), 2.23 (s, 3H); ^13^C NMR (126 MHz, CDCl_3_) δ 169.2, 142.8, 139.0, 137.2, 131.5, 118.7, 114.2, 71.7, 59.0, 48.8, 27.8, 24.3, 8.9. HRMS-ESI: [M + Na]^+^ calculated for C_16_H_23_BrN_2_NaO_5_S^+^, 457.0403; found, 457.0403. 

#### 4.5.19. 1-Acetyl-5-Bromo-*N*,*N*-Diethylindoline-6-Sulfonamide (**9n**)

Off-white solid (110 mg, 99%): mp 195-196 °C; ^1^H NMR (500 MHz, CDCl_3_) δ 8.72 (s, 1H), 7.47 (s, 1H), 4.13 (t, *J* = 8.5 Hz, 2H), 3.42 (q, *J* = 7.0 Hz, 4H), 3.23 (t, *J* = 8.0 Hz, 2H), 2.23 (s, 3H), 1.19 (t, *J* = 7.5 Hz, 6H); ^13^C NMR (126 MHz, CDCl_3_) δ 169.2, 142.7, 139.6, 136.9, 131.5, 118.1, 114.0, 49.2, 42.3, 27.7, 24.3, 14.4. HRMS-ESI: [M + Na]^+^ calculated for C_14_H_19_BrN_2_NaO_3_S^+^, 397.0192; found, 397.0186.

#### 4.5.20. 1-Acetyl-5-Chloro-*N*-Isopentylindoline-6-Sulfonamide (**10a**)

Tan solid (117 mg, 99%): mp 174 °C (softens), 179.1–180.4 °C; ^1^H NMR (300 MHz, CDCl_3_) δ 8.84 (s, 1H), 7.28 (s, 1H), 4.90 (t, *J* = 6.1 Hz, 1H), 4.14 (t, *J* = 8.6 Hz, 2H), 3.25 (t, *J* = 8.6 Hz, 2H), 2.93 (td, *J* = 7.3, 6.2 Hz, 2H), 2.24 (s, 3H), 1.60 (dq, *J* = 13.3, 6.6 Hz, 1H), 1.36 (q, *J* = 7.1 Hz, 2H), 0.84 (d, *J* = 6.6 Hz, 6H). ^13^C NMR (126 MHz, CDCl_3_) δ 167.8, 141.2, 136.2, 135.3, 126.3, 124.3, 117.8, 47.9, 40.6, 37.3, 26.7, 24.4, 23.0, 21.2. HRMS-ESI: [M + NH_4_]^+^ calculated for C_15_H_25_ClN_3_O_3_S^+^, 362.1305; found, 362.1301.

#### 4.5.21. 1-Acetyl-5-Chloro-*N*-Cyclohexylindoline-6-Sulfonamide (**10b**)

White solid (95.3 mg, 79%): mp 230.0–230.5 °C; ^1^H NMR (300 MHz, CDCl_3_) δ 8.9 (s, 1H), 7.28 (s, 1H), 4.90 (t, *J* = 6.0 Hz, 1H), 4.14 (t, *J* = 8.7 Hz, 2H), 3.25 (t, *J* = 8.7 Hz, 2H), 2.93 (dd, *J* = 13.7, 7.2 Hz, 2H), 2.24 (s, 3H), 1.36 (dd, *J* = 14.4, 6.9 Hz, 2H), 1.6 (m, 1H), 0.84 (d, *J* = 6.6 Hz, 6H). ^13^C NMR (126 MHz, CDCl_3_) δ 178.7, 168.8, 142.2, 137.9, 137.0, 127.3, 125.3, 118.4, 52.9, 48.9, 33.7, 27.7, 25.2, 24.5, 24.1. HRMS-ESI: [M + Na]^+^ calculated for C_16_H_21_ClN_2_NaO_3_S^+^, 379.0854; found, 379.0854.

#### 4.5.22. 1-(5-Chloro-6-(Piperidin-1-Ylsulfonyl)Indolin-1-yl)Ethan-1-One (**10c**)

Tan solid (104 mg, 79%): mp 199.7–201.4 °C; ^1^H NMR (500 MHz, CDCl_3_) δ 8.72 (s, 1H), 7.26 (s, 1H), 4.13 (t, *J* = 8.6 Hz, 2H), 3.28 (t, *J* = 5.4 Hz, 4H), 3.24 (t, *J* = 8.5 Hz, 2H), 2.23 (s, 3H), 1.63 (p, *J* = 5.6 Hz, 4H), 1.55 (t, *J* = 5.8 Hz, 2H). ^13^C NMR (126 MHz, CDCl_3_) δ 169.1, 142.1, 137.2, 135.9, 128.1, 126.6, 118.7, 49.1, 47.0, 27.9, 25.8, 24.2, 24.0. HRMS-ESI: [M + H]^+^ calculated for C_15_H_20_ClN_2_O_3_S^+^, 343.0878; found, 343.0871.

#### 4.5.23. 1-(5-Chloro-6-(Pyrrolidin-1-Ylsulfonyl)Indolin-1-yl)ethan-1-One (**10d**)

White crystalline solid (103 mg, 92%): mp 195 °C (softens), 199.7–200.4 °C; ^1^H NMR (500 MHz, CDCl_3_) δ 8.67 (s, 1H), 7.27 (s, 1H), 4.13 (t, *J* = 8.6 Hz, 2H), 3.44 (td, *J* = 6.7, 5.4, 2.9 Hz, 4H), 3.24 (t, *J* = 8.5 Hz, 2H), 2.23 (s, 3H), 1.99 – 1.93 (m, 4H). ^13^C NMR (126 MHz, CDCl_3_) δ 169.2, 142.1, 137.1, 136.4, 128.2, 126.6, 117.9, 49.2, 48.3, 27.9, 26.0, 24.3. HRMS-ESI: [M + Na]^+^ calculated for C_14_H_17_ClN_2_NaO_3_S^+^, 351.0541; found, 351.0537.

#### 4.5.24. 1-Acetyl-5-Chloro-*N*,*N*-Dipropylindoline-6-Sulfonamide (**10e**)

Light brown solid (104 mg, 85%): mp 118–120 °C; ^1^H NMR (500 MHz, CDCl_3_) δ 8.78 (s, 1H), 7.33 (s, 1H), 4.20 (t, *J* = 8.6 Hz, 2H), 3.37–3.32 (m, 4H), 3.30 (t, *J* = 8.5 Hz, 2H), 2.31 (s, 3H), 1.66 (h, *J* = 7.5 Hz, 4H), 0.92 (t, *J* = 7.4 Hz, 6H). ^13^C NMR (126 MHz, CDCl_3_) δ 169.2, 142.0, 137.8, 136.9, 128.0, 126.6, 118.0, 49.9, 49.6, 49.2, 27.9, 24.3, 22.1, 19.6, 11.5, 11.4. HRMS-ESI: [M + H]^+^ calculated for C_16_H_24_ClN_2_O_3_S^+^, 359.1191; found, 359.1187.

#### 4.5.25. 1-Acetyl-5-Chloro-*N*,*N*-bis(2-Methoxyethyl)Indoline-6-Sulfonamide (**10f**)

Gray solid (150 mg, 99%): mp 93.2–94.4 °C; ^1^H NMR (500 MHz, CDCl_3_) δ 8.77 (s, 1H), 7.27 (s, 1H), 4.13 (t, *J* = 8.6 Hz, 2H), 3.61–3.49 (m, 8H), 3.29 (s, 6H), 3.24 (t, *J* = 9.0 Hz, 3H), 2.23 (s, 3H). ^13^C NMR (126 MHz, CDCl_3_) δ 169.0, 142.0, 137.2, 137.1, 127.8, 126.5, 118.5, 71.6, 58.9, 49.1, 48.6, 27.8, 24.1. HRMS-ESI: [M + Na]^+^ calculated for C_16_H_23_ClN_2_NaO_5_S^+^, 413.0908; found, 413.0905.

## 5. Conclusions

In summary, we have synthesized a series of indoline-6-sulfonamide derivatives based on original hits discovered in a high-throughput screen, and we have assayed these compounds for inhibition of the bacterial enzyme DapE using our recently-reported ninhydrin-based assay. Docking experiments suggest that one of the electron-rich sulfonamide oxygens may serve as a ligand for one of the two Lewis acidic Zn(II) atoms in the active site of DapE. This study offers promise on the path toward drug-like small molecule inhibitors of DapE as antibiotics with a new mechanism of action.

## 6. Patents

Daniel P. Becker, Richard Holz, Tahirah Heath, Cory Reidl, and Anna Starus, “Indoline sulfonamide inhibitors of DapE and NDM-1 and use of the same” US 10,385,040, issued 8-20-19.

## Figures and Tables

**Table 1:**
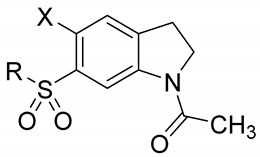
Inhibition of DapE enzyme by synthesized compounds **4**, **9a**–**n**, and **10a**–**f** ^a^.

0	X	R	MW	clogP	mp (°C)	IC_50_ (μM) or % Inhibition ^a^
**4**	Br	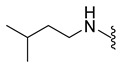	389.3	2.79	190–191	42% at 200 μM
**9a**	Br	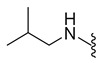	374.0	2.26	225.4–226.1	20% at 200 μM
**9b**	Br	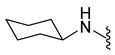	400.0	2.84	226–228	162
**9c**	Br	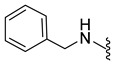	408.0	2.57	205–208	56% at 200 μM
**9d**	Br	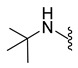	374.0	2.04	235.7–236.1	39% at 100 μM
**9e**	Br	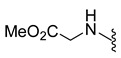	390.0	0.79	179–182	17% at 200 μM
**9f**	Br	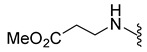	404.0	1.1	190–193	118
**9g**	Br	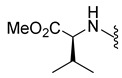	432.0	2.0	190.9–191.7	82
**9h**	Br	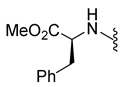	480.0	2.5	184.0–184.8	61% at 200 μM
**9i**	Br	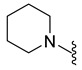	386.0	2.1	212–214	133
**9j**	Br	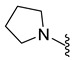	372.0	1.6	238–240	97
**9k**	Br	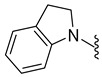	420.0	2.6	215–218	86
**9l**	Br	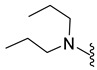	402.1	3.0	118–120	5% at 20 μM, insol. at 200 μM
**9m**	Br	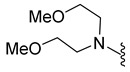	434.1	1.5	110–111	26% at 100 μM
**9n**	Br	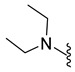	374.0	2.0	195–196	99
**10a**	Cl	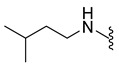	344.1	2.8	179.1–180.4	54
**10b**	Cl	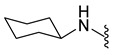	356.1	2.9	230.0–230.5	58% at 200 μM
**10c**	Cl	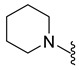	342.1	2.2	199.7–201.4	44
**10d**	Cl	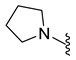	328.1	1.6	199.7–200.4	172
**10e**	Cl	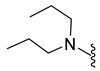	358.1	3.1	118–120	88
**10f**	Cl	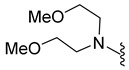	390.1	1.5	93.2–94.4	>200

^a^ IC_50_ values were determined from a set of three or four % inhibition points, and percent inhibition values were determined at a single concentration; insol. = insoluble.

## Supplementary Materials

The following are available online at https://www.mdpi.com/2079-6382/9/9/595/s1, Figure S1: copies of spectral characterization of compounds **4**, **7b**, **8a**, **8b**, **9a**–**n**, and **10a**–**f**.
