# Supplementary material for: Indoline-6-Sulfonamide Inhibitors of the Bacterial Enzyme DapE
Source: Antibiotics (Basel). 2020 Sep 11;9(9):595. doi: 10.3390/antibiotics9090595 (PMC7560015; doi:10.3390/antibiotics9090595)
Supplement: Supplementary file 1 [file antibiotics-09-00595-s001.zip › antibiotics-913614-supplementary.docx]

| **Table of Contents** | |  |
| --- | --- | --- |
| **Figures S1.** copies of spectral characterization of compounds. |  | |
| **SI-Fig. 1.** ^1^H NMR (500 MHz CDCl_3_) for 1-Acetyl-5-chloroindoline (**7b**). | S4 | |
| **SI-Fig. 2.** ^13^C NMR (126 MHz, CDCl_3_) for 1-Acetyl-5-chloroindoline (**7b**). | S5 | |
| **SI-Fig. 3**. Laboratory apparatus setup for continuous flow chlorination synthesis of **7b**. | S6 | |
| **SI-Fig. 4.** ^1^H NMR (500 MHz CDCl_3_) for 1-Acetyl 5-bromoindoline -6-sulfonyl chloride (**8a**). | S7 | |
| **SI-Fig. 5.** ^13^C NMR (126 MHz, (DMSO-*d*_6_) for 1-Acetyl 5-bromoindoline -6-sulfonyl chloride (**8a**). | S8 | |
| **SI-Fig. 6.** ^1^H NMR (500 MHz, DMSO-*d*_6_) for 1-Acetyl-5-chloroindoline-6-sulfonyl chloride (**8b**). | S9 | |
| **SI-Fig. 7.** ^1^H NMR (500 MHz, CDCl_3_) for 1-Acetyl-5-bromo-*N*-isopentylindoline-6-sulfonamide (**4**). | S10 | |
| **SI-Fig. 8.** ^13^C NMR (126 MHz, CDCl_3_) for 1-Acetyl-5-bromo-*N*-isopentylindoline-6-sulfonamide (**4**). | S11 | |
| **SI-Fig. 9.** ^1^H NMR (500 MHz, CDCl_3_) for 1-Acetyl-5-bromo-*N*-isobutylindoline-6-sulfonamide (**9a**). | S12 | |
| **SI-Fig. 10.** ^13^C NMR (126 MHz, CDCl_3_) for 1-Acetyl-5-bromo-*N*-isobutylindoline-6-sulfonamide (**9a**). | S13 | |
| **SI-Fig. 11.** ^1^H NMR (500 MHz, CDCl_3_) for 1-Acetyl-5-bromo-*N*-cyclohexylindoline-6-sulfonamide (**9b**). | S14 | |
| **SI-Fig. 12.** ^13^C NMR (126 MHz, CDCl_3_) for 1-Acetyl-5-bromo-*N*-cyclohexylindoline-6-sulfonamide (**9b**). | S15 | |
| **SI-Fig. 13.** ^1^H NMR (500 MHz, CDCl_3_) for 1-Acetyl-*N*-benzyl-5-bromoindoline-6-sulfonamide (**9c**). | S16 | |
| **SI-Fig. 14.** ^1^H NMR (500 MHz, CDCl_3_) for 1-Acetyl-5-bromo-*N*-(tert-butyl)indoline-6-sulfonamide (**9d**). | S17 | |
| **SI-Fig. 15.** ^13^C NMR (126 MHz, CDCl_3_) for 1-Acetyl-5-bromo-*N*-(tert-butyl)indoline-6-sulfonamide (**9d**). | S18 | |
| **SI-Fig. 16.** ^1^H NMR (500 MHz, CDCl_3_) for 1-Acetyl-5-bromoindolin-6-(sulfonyl glycine methyl ester) (**9e**). | S19 | |
| **SI-Fig. 17.** ^13^C NMR (126 MHz, CDCl_3_) for 1-Acetyl-5-bromoindolin-6-(sulfonyl glycine methyl ester) (**9e**). | S20 | |
| **SI-Fig. 18.** ^1^H NMR (500 MHz, CDCl_3_) for Methyl 3-((1-acetyl-5-bromoindoline)-6-sulfonamido)propanoate **(9f).** | S21 | |
| **SI-Fig. 19.** ^13^C NMR (126 MHz, CDCl_3_) for Methyl 3-((1-acetyl-5-bromoindoline)-6-sulfonamido)propanoate **(9f).** | S22 | |
| **SI-Fig. 20.** ^1^H NMR (500 MHz, CDCl_3_) for Methyl ((1-acetyl-5-bromoindolin-6-yl)sulfonyl)valinate (**9g**). | S23 | |
| **SI-Fig. 21.** ^13^C NMR (126 MHz, CDCl_3_) for Methyl ((1-acetyl-5-bromoindolin-6-yl)sulfonyl)valinate (**9g**). | S24 | |
| **SI-Fig. 22.** ^1^H NMR (300 MHz, CDCl_3_) for Methyl ((1-acetyl-5-bromoindolin-6-yl)sulfonyl)-L-phenylalaninate (**9h**). | S25 | |
| **SI-Fig. 23.** ^13^C NMR (126 MHz, CDCl_3_) for Methyl ((1-acetyl-5-bromoindolin-6-yl)sulfonyl)-L-phenylalaninate (**9h**). | S26 | |
| **SI-Fig. 24.** ^1^H NMR (500 MHz, CDCl_3_) for 1-Acetyl-5-bromo-6-(piperidin-1-sulfonyl) indoline (**9i**). | S27 | |
| **SI-Fig. 25.** ^13^C NMR (126 MHz, CDCl_3_) for 1-Acetyl-5-bromo-6-(piperidin-1-sulfonyl) indoline (**9i**). | S28 | |
| **SI-Fig. 26.** ^1^H NMR (500 MHz, CDCl_3_) for 1-Acetyl-5-bromo-6-(pyrrolidin-1-sulfonyl) indoline (**9j**). | S29 | |
| **SI-Fig. 27.** ^13^C NMR (126 MHz, CDCl_3_) for 1-Acetyl-5-bromo-6-(pyrrolidin-1-sulfonyl) indoline (**9j**). | S30 | |
| **SI-Fig. 28.** ^1^H NMR (500 MHz, CDCl_3_) for 1-Acetyl-5-bromo-6-(indolin-1-sulfonyl) indoline (**9k**). | S31 | |
| **SI-Fig. 29.** ^1^H NMR (500 MHz, CDCl_3_) for 1-Acetyl-5-bromo-*N*,*N*-dipropylindoline-6-sulfonamide (**9l**). | S32 | |
| **SI-Fig. 30.** ^1^H NMR (500 MHz, CDCl_3_) for 1-Acetyl-5-bromo-*N*,*N*-bis(2-methoxyethyl)indoline-6-sulfonamide (**9m**). | S33 | |
| **SI-Fig. 31.** ^13^C NMR (126 MHz, CDCl_3_) for **1**-Acetyl-5-bromo-*N*,*N*-bis(2-methoxyethyl)indoline-6-sulfonamide (**9m**). | S34 | |
| **SI-Fig. 32.** ^1^H NMR (500 MHz, CDCl_3_) for 1-Acetyl-5-bromo-*N*,*N*-diethylindoline-6-sulfonamide (**9n**). | S35 | |
| **SI-Fig. 33.** ^13^C NMR (126 MHz, CDCl_3_) for 1-Acetyl-5-bromo-*N*,*N*-diethylindoline-6-sulfonamide (**9n**). | S36 | |
| **SI-Fig. 34.** ^1^H NMR (300 MHz, CDCl_3_) for 1-Acetyl-5-chloro-*N*-isopentylindoline-6-sulfonamide (**10a**). | S37 | |
| **SI-Fig. 35.** ^13^C NMR (126 MHz, CDCl_3_) for 1-Acetyl-5-chloro-*N*-isopentylindoline-6-sulfonamide **(10a).** | S38 | |
| **SI-Fig. 36.** ^1^H NMR (300 MHz, CDCl_3_) for 1-Acetyl-5-chloro-*N*-cyclohexylindoline-6-sulfonamide (**10b**). | S39 | |
| **SI-Fig. 37.** ^13^C NMR (126 MHz, CDCl_3_) for 1-Acetyl-5-chloro-*N*-cyclohexylindoline-6-sulfonamide (**10b**). | S40 | |
| **SI-Fig. 38.** ^1^H NMR (500 MHz, CDCl_3_) for 1-(5-Chloro-6-(piperidin-1-ylsulfonyl)indolin-1-yl)ethan-1-one (**10c**). | S41 | |
| **SI-Fig. 39.** ^13^C NMR (126 MHz, CDCl_3_) for 1-(5-Chloro-6-(piperidin-1-ylsulfonyl)indolin-1-yl)ethan-1-one (**10c**). | S42 | |
| **SI-Fig. 40.** ^1^H NMR (500 MHz, CDCl_3_) for 1-(5-Chloro-6-(pyrrolidin-1-ylsulfonyl)indolin-1-yl)ethan-1-one (**10d**). | S43 | |
| **SI-Fig. 41.** ^13^C NMR (126 MHz, CDCl_3_) for 1-(5-Chloro-6-(pyrrolidin-1-ylsulfonyl)indolin-1-yl)ethan-1-one (**10d**). | S44 | |
| **SI-Fig. 42.** ^1^H NMR (500 MHz, CDCl_3_) for 1-Acetyl-5-chloro-*N*,*N*-dipropylindoline-6-sulfonamide (**10e**). | S45 | |
| **SI-Fig. 43.** ^13^C NMR (126 MHz, CDCl_3_) for 1-Acetyl-5-chloro-*N,N*-dipropylindoline-6-sulfonamide (**10e**). | S46 | |
| **SI-Fig. 44.** ^1^H NMR (500 MHz, CDCl_3_) for 1-Acetyl-5-chloro-*N*,*N*-bis(2-methoxyethyl)indoline-6-sulfonamide (**10f**). | S47 | |
| **SI-Fig. 45.** ^13^C NMR (126 MHz, CDCl_3_) for 1-Acetyl-5-chloro-*N,N*-bis(2-methoxyethyl)indoline-6-sulfonamide(**10f**). | S48 | |
|  |  | |

|  |
| --- |
| **(7b)** |

**SI-Figure 1.** ^1^H NMR (500 MHz CDCl_3_) for 1-Acetyl-5-chloroindoline (**7b**).

**(7b)**

**SI-Figure 2.** ^13^C NMR (126 MHz, CDCl_3_) for 1-Acetyl-5-chloroindoline (**7b**).

**
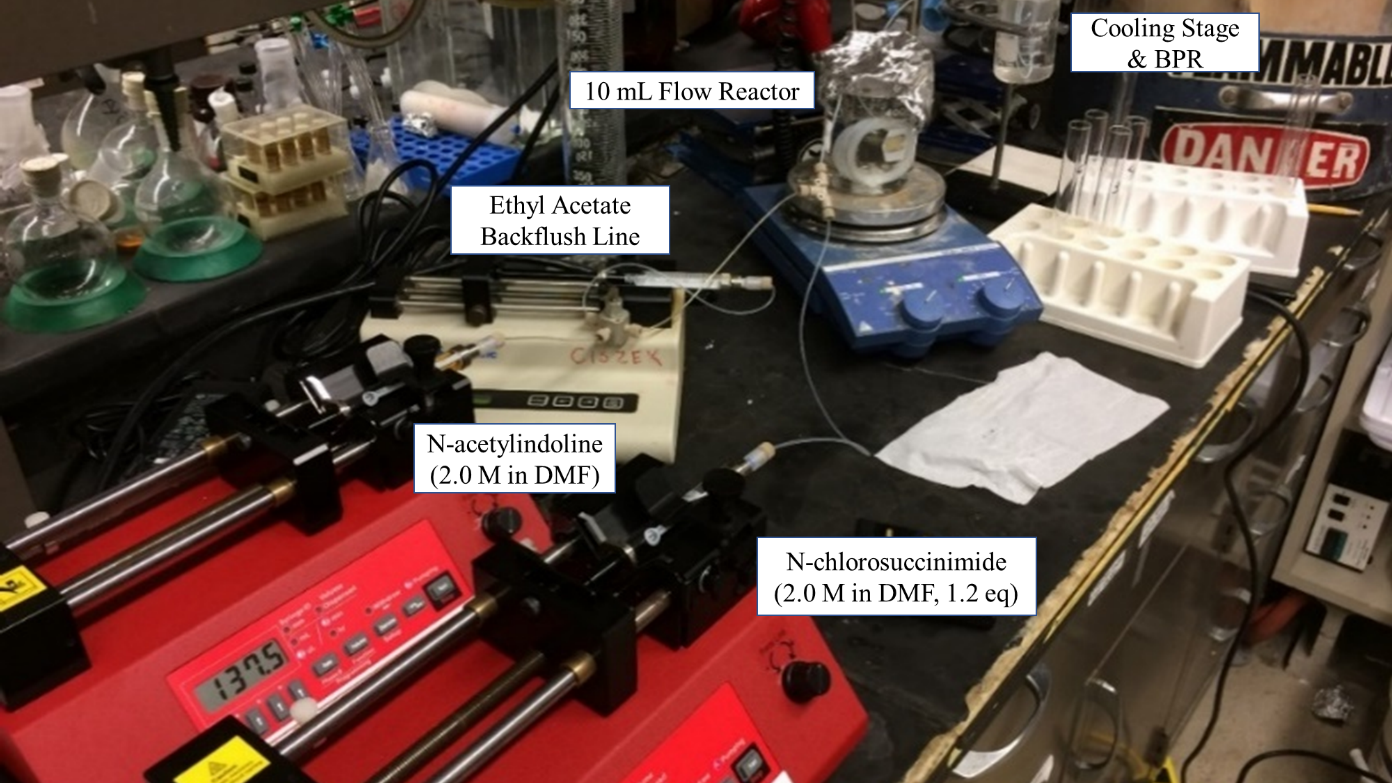
**

**SI-Figure 3**. Flow apparatus for continuous chlorination providing **7b**.

For reference in relationship to SI-Figure 3 directly above, here follows a copy of Figure 4 from the manuscript.

**Manuscript Figure 4.** Flow chemistry apparatus for preparation of *N*-acetyl-5-chloroindoline **7b**

|  |
| --- |
| **(8a)** |

**SI-Figure 4.** ^1^H NMR (500 MHz CDCl_3_) for 1-Acetyl 5-bromoindoline-6-sulfonyl chloride (**8a**).

| **(8a)** |
| --- |

**SI-Figure 5.** ^13^C NMR (126 MHz, DMSO-*d*_6_) for 1-Acetyl 5-bromoindoline-6-sulfonyl chloride (**8a**).

|  |
| --- |
| **(8b)** |

**SI-Figure 6.** ^1^H NMR (500 MHz, DMSO-*d*_6_) for 1-Acetyl-5-chloroindoline-6-sulfonyl chloride (**8b**).

|  |
| --- |
| **(4)** |

**SI-Figure 7.** ^1^H NMR (500 MHz, CDCl_3_) for 1-Acetyl-5-bromo-*N*-isopentylindoline-6-sulfonamide **(4**).

**SI-Figure 8.** ^13^C NMR (126 MHz, CDCl_3_) for 1-Acetyl-5-bromo-*N*-isopentylindoline-6-sulfonamide **(4**).

|  |
| --- |
| **(9a)** |

**SI-Figure 9.** ^1^H NMR (500 MHz, CDCl_3_) for 1-Acetyl-5-bromo-*N*-isobutylindoline-6-sulfonamide (**9a**).

| **** |
| --- |
| **(9a)** |

**SI-Figure 10.** ^13^C NMR (126 MHz, CDCl_3_) for 1-Acetyl-5-bromo-*N*-isobutylindoline-6-sulfonamide (**9a**).

|  |
| --- |
| **(9b)** |

**SI-Figure 11.** ^1^H NMR NMR (500 MHz, CDCl_3_) for 1-Acetyl-5-bromo-*N*-cyclohexylindoline-6-sulfonamide (**9b**).

| **** |
| --- |
| **(9b)** |

**SI-Figure 12.** ^13^C NMR (126 MHz, CDCl_3_) for 1-Acetyl-5-bromo-*N*-cyclohexylindoline-6-sulfonamide (**9b**).

|  |
| --- |
| **(9c)** |

**SI-Figure 13.** ^1^H NMR (500 MHz, CDCl_3_) for 1-Acetyl-*N*-benzyl-5-bromoindoline-6-sulfonamide (**9c**).

|  |
| --- |
| **(9d)** |
|  |

**SI-Figure 14.** ^1^H NMR (500 MHz, CDCl_3_) for 1-Acetyl-5-bromo-*N*-(tert-butyl)indoline-6-sulfonamide (**9d**).

|  |
| --- |
| **(9d)** |

**SI-Figure 15.** ^13^C NMR (126 MHz, CDCl_3_) for 1-Acetyl-5-bromo-*N*-(tert-butyl)indoline-6-sulfonamide (**9d**).

|  |
| --- |
| **(9e)** |

**SI-Figure 16.** ^1^H NMR (500 MHz, CDCl_3_) for 1-Acetyl-5-bromoindolin-6-(sulfonyl glycine methyl ester) (**9e**).

|  |
| --- |
| **(9e)** |

**SI-Figure 17.** ^13^C NMR (126 MHz, CDCl_3_) for 1-Acetyl-5-bromoindolin-6-(sulfonyl glycine methyl ester) (**9e**).

|  |
| --- |
| **(9f)** |

**SI-Figure 18.** ^1^H NMR (500 MHz, CDCl_3_) for Methyl 3-((1-acetyl-5-bromoindoline)-6-sulfonamido)propanoate (**9f**).

|  |
| --- |
| **(9f)** |

**SI-Figure 19.** ^13^C NMR (126 MHz, CDCl_3_) for Methyl 3-((1-acetyl-5-bromoindoline)-6-sulfonamido)propanoate (**9f**).

|  |
| --- |
| **(9g)** |

**SI-Figure 20.** ^1^H NMR (300 MHz, CDCl_3_) for Methyl ((1-acetyl-5-bromoindolin-6-yl)sulfonyl)valinate (**9g**).

|  |
| --- |
| **(9g)** |

**SI-Figure 21.** ^13^C NMR (126 MHz, CDCl_3_) for Methyl ((1-acetyl-5-bromoindolin-6-yl)sulfonyl)valinate (**9g**).

|  |
| --- |
| **(9h)** |

**SI-Figure 22.** ^1^H NMR (300 MHz, CDCl_3_) for Methyl ((1-acetyl-5-bromoindolin-6-yl)sulfonyl)-L-phenylalaninate (**9h**).

|  |
| --- |
| **(9h)** |

**SI-Figure 23.** ^13^C NMR (126 MHz, CDCl_3_) for Methyl ((1-acetyl-5-bromoindolin-6-yl)sulfonyl)-L-phenylalaninate (**9h**).

|  |
| --- |
| **(9i)** |

**SI-Figure 24.** ^1^H NMR (500 MHz, CDCl_3_) for 1-Acetyl-5-bromo-6-(piperidin-1-sulfonyl) indoline (**9i**).

|  |
| --- |
| **(9i)** |

**SI-Figure 25.** ^13^C NMR (126 MHz, CDCl_3_) for 1-Acetyl-5-bromo-6-(piperidin-1-sulfonyl) indoline (**9i**).

|  |
| --- |
| **(9j)** |

**SI-Figure 26.** ^1^H NMR (500 MHz, CDCl_3_) for 1-Acetyl-5-bromo-6-(pyrrolidin-1-sulfonyl) indoline (**9j**).

| **** |
| --- |
| **(9j)** |

**SI-Figure 27.** ^13^C NMR (126 MHz, CDCl_3_) for 1-Acetyl-5-bromo-6-(pyrrolidin-1-sulfonyl) indoline (**9j**).

|  |
| --- |
| **(9k)** |

**SI-Figure 28.** ^1^H NMR (500 MHz, CDCl_3_) for 1-Acetyl-5-bromo-6-(indolin-1-sulfonyl) indoline (**9k**).

|  |
| --- |
| **(9l)** |

**SI-Figure 29.** ^1^H NMR (500 MHz, CDCl_3_) for 1-Acetyl-5-bromo-*N*,*N*-dipropylindoline-6-sulfonamide (**9l**).

|  |
| --- |
| **(9m)** |

**SI-Figure 30.** ^1^H NMR (500 MHz, CDCl_3_) for 1-Acetyl-5-bromo-*N*,*N*-bis(2-methoxyethyl)indoline-6-sulfonamide (**9m**).

| **** |
| --- |
| **(9m)** |

**SI-Figure 31.** ^13^C NMR (126 MHz, CDCl_3_) for 1-Acetyl-5-bromo-*N*,*N*-bis(2-methoxyethyl)indoline-6-sulfonamide (**9m**).

|  |
| --- |
| **(9n)** |

**SI-Figure 32.** ^1^H NMR (500 MHz, CDCl_3_) for 1-Acetyl-5-bromo-*N*,*N*-diethylindoline-6-sulfonamide (**9n**).

|  |
| --- |
| **(9n)** |

**SI-Figure 33.** ^13^C NMR (126 MHz, CDCl_3_) for 1-Acetyl-5-bromo-*N*,*N*-diethylindoline-6-sulfonamide (**9n**).

|  |
| --- |
| **(10a)** |

**SI-Figure 34.** ^1^H NMR (300 MHz, CDCl_3_) for 1-Acetyl-5-chloro-*N*-isopentylindoline-6-sulfonamide **(10a).**

| **** |
| --- |
| **(10a)** |

**SI-Figure 35.** ^13^C NMR (126 MHz, CDCl_3_) for 1-Acetyl-5-chloro-*N*-isopentylindoline-6-sulfonamide (**10a**).

| \| **** \| \| --- \| \| **(10b)** \| |
| --- | --- | --- |

**SI-Figure 36.** ^1^H NMR (300 MHz, CDCl_3_) for 1-Acetyl-5-chloro-*N*-cyclohexylindoline-6-sulfonamide (**10b**).

| **** |
| --- |
| **(10b)** |

**SI-Figure 37.** ^13^C NMR (126 MHz, CDCl_3_) for 1-Acetyl-5-chloro-*N*-cyclohexylindoline-6-sulfonamide (**10b**).

| \| **** \| \| --- \| \| **(10c)** \| |
| --- | --- | --- |

**SI-Figure 38.** ^1^H NMR (500 MHz, CDCl_3_) for 1-(5-Chloro-6-(piperidin-1-ylsulfonyl)indolin-1-yl)ethan-1-one (**10c**).

| **** |
| --- |
| **(10c)** |

**SI-Figure 39.** ^13^C NMR (126 MHz, CDCl_3_) for 1-(5-Chloro-6-(piperidin-1-ylsulfonyl)indolin-1-yl)ethan-1-one (**10c**).

| **** |
| --- |
| **(10d)** |

**SI-Figure 40.** ^1^H NMR (500 MHz, CDCl_3_) for 1-(5-Chloro-6-(pyrrolidin-1-ylsulfonyl)indolin-1-yl)ethan-1-one (**10d**).

| **** |
| --- |
| **(10d)** |

**SI-Figure 41.** ^13^C NMR (126 MHz, CDCl_3_) for 1-(5-Chloro-6-(pyrrolidin-1-ylsulfonyl)indolin-1-yl)ethan-1-one (**10d**).

|  |
| --- |
| **(10e)** |

**SI-Figure 42.** ^1^H NMR (500 MHz, CDCl_3_) for 1-Acetyl-5-chloro-*N*,*N*-dipropylindoline-6-sulfonamide (**10e**).

|  |
| --- |
| **(10e)** |

**SI-Figure 43.** ^13^C NMR (126 MHz, CDCl_3_) for 1-Acetyl-5-chloro-*N*,*N*-dipropylindoline-6-sulfonamide (**10e**).

|  |
| --- |
| **(10f)** |

 **SI-Figure 44.** ^1^H NMR (500 MHz, CDCl_3_) for 1-Acetyl-5-chloro-*N*,*N*-bis(2-methoxyethyl)indoline-6-sulfonamide (**10f**).

|  |
| --- |
| **(10f)** |

**SI-Figure 45.** ^13^C NMR (126 MHz, CDCl_3_) for 1-Acetyl-5-chloro-*N*,*N*-bis(2-methoxyethyl)indoline-6-sulfonamide (**10f**).
